# A Randomized Longitudinal Factorial Design to Assess Malaria Vector Control and Disease Management Interventions in Rural Tanzania

**DOI:** 10.3390/ijerph110505317

**Published:** 2014-05-16

**Authors:** Randall A. Kramer, Leonard E. G. Mboera, Kesheni Senkoro, Adriane Lesser, Elizabeth H. Shayo, Christopher J. Paul, Marie Lynn Miranda

**Affiliations:** 1 Duke Global Health Institute, Duke University, 310 Trent Drive, Durham, NC 27710, USA; E-Mails: kramer@duke.edu (R.A.K.); adriane.lesser@duke.edu (A.L.); 2 Nicholas School of the Environment, Duke University, 9 Circuit Drive, Durham, NC 27708, USA; E-Mail: christopher.paul@duke.edu; 3 National Institute for Medical Research, 2448 Barack Obama Drive, P.O. Box 9653, Dar es Salaam, United Republic of Tanzania; E-Mails: lmboera@nimr.or.tz (L.E.G.M.); ksenkoro@nimr.or.tz (K.S.); eshayo@nimr.or.tz (E.H.S.); 4 School of Natural Resources and Environment, University of Michigan, 440 Church Street, Ann Arbor, MI 48109, USA; 5 Departments of Pediatrics and Obstetrics & Gynecology, School of Medicine, University of Michigan, Ann Arbor, MI 48109, USA

**Keywords:** malaria, disease management, vector control, larviciding, factorial design, implementation science, community health delivery experiments, Tanzania

## Abstract

The optimization of malaria control strategies is complicated by constraints posed by local health systems, infrastructure, limited resources, and the complex interactions between infection, disease, and treatment. The purpose of this paper is to describe the protocol of a randomized factorial study designed to address this research gap. This project will evaluate two malaria control interventions in Mvomero District, Tanzania: (1) a disease management strategy involving early detection and treatment by community health workers using rapid diagnostic technology; and (2) vector control through community-supported larviciding. Six study villages were assigned to each of four groups (control, early detection and treatment, larviciding, and early detection and treatment plus larviciding). The primary endpoint of interest was change in malaria infection prevalence across the intervention groups measured during annual longitudinal cross-sectional surveys. Recurring entomological surveying, household surveying, and focus group discussions will provide additional valuable insights. At baseline, 962 households across all 24 villages participated in a household survey; 2,884 members from 720 of these households participated in subsequent malariometric surveying. The study design will allow us to estimate the effect sizes of different intervention mixtures. Careful documentation of our study protocol may also serve other researchers designing field-based intervention trials.

## 1. Introduction

An estimated 207 million cases of malaria and 627,000 malaria deaths occurred globally in 2012 [1]. Approximately 90% of these deaths were in sub-Saharan Africa, and 77% of deaths attributed to malaria globally occurred among children under five years of age [1]. In 33 countries of sub-Saharan Africa, malaria is the cause of greater than 10% of deaths among children under five years of age [1]. There were an estimated 11.5 million clinical malaria cases in mainland Tanzania in 2008, and the National Malaria Control Programme has estimated that there are 60,000–80,000 malaria-related deaths there annually [2]. A widely cited study by Gallup and Sachs [3] estimated that malaria was responsible for lowering per-capita economic growth by 1.3% per year in malarial areas, controlling for other factors. While the precision of these estimates is debatable, due to the limited availability and reliability of data on confirmed malaria diagnoses [4], malaria clearly imposes a substantial social and economic burden, especially in sub-Saharan Africa. 

In 2012, international and domestic sources combined committed an estimated 2.5 billion USD in financing for malaria control, including support for both disease prevention and management strategies [1]. Yet it can be hard to predict the impact of malaria control interventions, in part because of the complicated interactions between infection, disease, and treatment. Decision-makers operating in malaria endemic countries also face significant challenges in determining the best combination of control strategies to implement given the constraints posed by local health systems, infrastructure, and limited resources. 

Malaria control strategies can include two very different sets of approaches: treating the disease or treating the vector. The former seeks to minimize the impact of infection, and the latter aims to reduce transmission and hence acquisition of the infection. Treating the disease includes the use of anti-malarial drugs and reliable and sensitive diagnostic methods. In many areas of the world, resistance to anti-malarial medications has emerged [5,6], making good diagnostic methods yet more important. More reliable diagnostic methods can help ensure that only true malaria cases are treated with anti-malarial drugs, allowing more expensive frontline medicines such as artemisinin-combination therapy (ACT) to be used more effectively [7]. Rapid diagnostic testing (RDT) verifies infection in patients exhibiting symptoms, usually fever. The beneficial impacts of RDT may include a more efficient use of finite resources, through enhanced health outcomes and reduced costs, as well as a diminished likelihood of resistance to ACT [8,9]. 

Vector control measures are an important component of malaria prevention strategies and may include larval source management (LSM), indoor residual spraying (IRS), and the use of insecticide-treated mosquito nets (ITNs). However, the integration of the interventions, integrated vector management (IVM), is considered the most effective approach [10,11]. ITNs and IRS are used most commonly, although the latter remains controversial, particularly because of its association with DDT use [12,13]. The use of microbial larvicides (an LSM approach) has been found to be safe and effective at reducing vector populations and shows considerable potential as a part of a successful IVM strategy [14,15]. Biological control using biolarvicides is included in the current Tanzania Medium Term Malaria Strategic Plan [2]. While recent research in Dar es Salaam demonstrates the feasibility and potential of larviciding in an urban area [16], less is known about its use in rural settings [17]. Moreover, more research is needed to determine the impact of larviciding on malaria incidence.

Most health improving interventions in low-income countries are not fully tested because they have not been evaluated under real world field conditions. As a result, there is a striking gap between health innovations and their delivery to low-income countries [18]. While evidence-based research through randomized trials is standard practice for evaluating drug interventions, the use of field experiments to evaluate health delivery has been much less common [19,20,21]. Resource poor communities face an array of constraints and multiple health threats that make it difficult to implement and sustain effective interventions [19,20]. 

There is growing recognition that an implementation science approach to health delivery is needed to improve health outcomes. Health delivery experiments can provide data on the additive and interactive effects of multiple policy interventions under actual field conditions [22]. The purpose of this paper is to present our study protocol, which was designed to assess the effects and effectiveness of relevant, locally-feasible health delivery interventions implemented under realistic field conditions through experiments and program evaluation methods.

There are a number of reasons we present this protocol design in advance of availability of final results. First, presenting our study protocol provides a forum to share in a more timely manner the approach and methods of our study design, which we believe will be of interest to others involved in implementation science research, as well as the global health research community more broadly. Second, making our protocol available may also reduce the potential for unintended duplication of our activities and allow for earlier exchanges and collaborations with others. The publication of study protocols has also been cited as a mechanism for increasing transparency and higher standards in research [23]. 

### 1.1. Objectives and Hypothesis

The primary objective of the health delivery experiments was to evaluate the role of disease management (home-based management consisting of early detection and treatment), vector management (larviciding), and these two interventions in combination, in malaria control. Specifically, the study sought to test the hypothesis that home management of malaria through early detection by community health workers (CHWs) using rapid diagnostic tests and prompt treatment, and vector management through larviciding by community applicators, used independently, or in combination, result in differential potential reduction of the malaria burden in rural villages of Mvomero district in Tanzania. The secondary objectives were to evaluate baseline and follow-up socio-economic conditions and attitudes, evaluate baseline and post intervention parasitemia levels, and evaluate entomological conditions.

### 1.2. Outcomes

The key outcome of interest was malaria burden compared across study arms and years. We approximated changes in malaria cases based on the presence of parasitemia, measured during annual cross-sectional malariometric surveys. Because young children are particularly vulnerable to malaria, the primary endpoint of interest was parasitemia measured in children under the age of five years old in households within the villages. The secondary outcomes were to assess any changes in parasitemia among the whole study population by study arm and year, to assess anemia levels in the study population, and to assess a number of entomological outcomes. 

## 2. Experimental Section

### 2.1. Study Area

**Figure 1:**
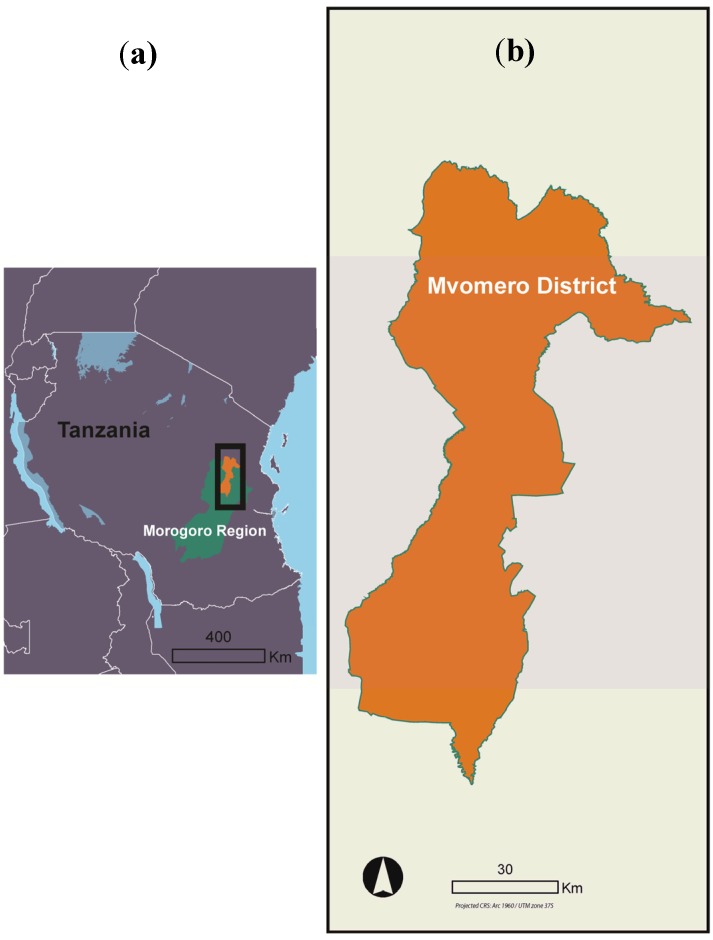
A map of (**a**) Tanzania and (**b**) Mvomero district.

This study was done across 24 villages located in six wards within the northern part of Mvomero district (Mvomero and Turiani divisions), Morogoro region, Tanzania (Figure 1), which is located between latitudes 6–10°S and longitudes 28–37°E, between 293 and 379 meters above sea level, with a total area of 7,325 km^2^. The district lies on the foothills of Nguru Mountains to the north-west and Uluguru Mountains to the south-east and falls within the Wami River Basin. The study area experiences two rainy seasons running from approximately March through May and October through December, and the average annual rainfall is high at 1,146 mm (the mean from data collected from 1953‒2003), though there is considerable variability across the study area. Malaria is endemic to the study area, with average prevalence in the district estimated at 34.5% in 2005 [24]. In Mvomero district, most adults (80%) are involved in largely subsistence agricultural activities, including rice, maize, and sugarcane production [25]. 

### 2.2. Study Design

**Figure 2:**
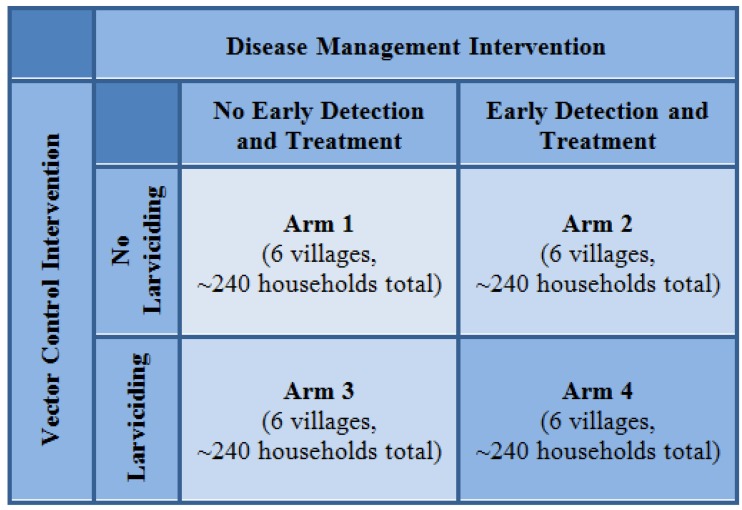
Factorial study design.

To implement an evaluation of health delivery experiments, the project conducted a large cluster-randomized health delivery experiment in 24 villages. The study sought to evaluate one vector management intervention (larviciding) and one disease treatment intervention (home-based management of malaria consisting of early RDT detection and treatment by village health workers). To assess the individual and combined effects of these two interventions, a 2 × 2 factorial design was developed with six villages randomly assigned to each of four study arms: (1) control/no intervention; (2) early detection and treatment; (3) larviciding intervention; and (4) both early detection and treatment and larviciding (Figure 2). In order to evaluate the effects and effectiveness of these health interventions, household- and individual-level data were collected before and after implementation of the health delivery experiments using household, entomological, and malariometric surveys in addition to local staff reporting on health delivery experiment implementation. 

#### 2.2.1. Household Knowledge, Attitude, and Practices Survey

Project investigators and researchers designed the household knowledge, attitudes, and practices (KAP) survey through an iterative process which included piloting a near-final draft within a representative population in Kibaha district (outside of the study area). Household survey data was then collected from approximately 40 randomly-selected households in each of the 24 villages. NIMR selected, trained, and closely supervised a team of experienced researchers who conducted face-to-face interviews with one adult member per household. The interviews sought information on household member characteristics; fever history and management; knowledge, attitudes, and practices regarding malaria; and awareness and level of acceptance towards RDTs and larviciding. 

#### 2.2.2. Focus Group Discussions and In-depth Interviews

Experienced social scientists also conducted focus group discussions (FGDs) and in-depth interviews (IDIs) in Kiswahili. FGD and IDI guides were developed collaboratively by study researchers and were also piloted in Kibaha district. IDI participants were key informants including village chairmen, village health workers and village executive officers. The FGDs and IDIs aimed at getting participants’ perspectives on community knowledge and behaviors related to malaria. Household surveying, FGDs, and IDIs were conducted in subsequent year(s) of the study in modified formats (household surveying in both of the subsequent years, and FGDs and IDIs in the final year of the study). 

#### 2.2.3. Malariometric Surveying

Malariometric assessments were carried out in all 24 study villages in each of the three years (baseline and two intervention years). Eligible participants consisted of any member of an enrolled study household. On an appointed day, collection occurred at a central data collection point facility in each village (generally a health facility, but a primary school on a few occasions). Villages with relatively low participation were re-visited on a second date. For each participant, basic demographic information (name, sex, age) was recorded along with axillary temperature, malaria treatment history in the past two weeks, height, and weight. In addition, arm circumference was recorded for all participants under the age of five, and presence or absence of splenomegaly was determined by a project physician for all participants under 15 years of age. Trained technicians obtained blood samples with a lancet for assessing anemia (HGB (g/L)), a thick and thin smear for later microscopic evaluation for malaria parasites, and the collection of dried blood spots on filter paper cards for later additional serological analyses.

#### 2.2.4. Entomological Sampling

The entomological sampling was carried in separate rounds spaced at regular intervals both before and after the introduction of interventions to measure impacts. Each round involved adult mosquito collections in each of three sentinel houses per village using a CDC light trap and pyrethrum spray catch methods. Mosquito collection from the CDC light trap was conducted for each sentinel household on three consecutive days, and pyrethrum spray catch was performed in addition to the CDC light trap collection on one day in each of the sentinel houses. Sentinel houses in the same village were sampled on the same three consecutive nights, but villages were sampled in sequential groups determined by proximity to one another (*i.e.*, entomological surveying did not occur in all 24 villages on the same three nights). A field laboratory was used to sort, identify species and count collected mosquitoes. The transmission parameters considered included anopheline species composition and relative density, parity of female mosquitoes from a sample of unfed mosquitoes, proportion of mosquitoes fed on human blood, and sporozoite rates. 

#### 2.2.5. District and Community Engagement Strategy

Community sensitization meetings were held in each study village in each year of the project to inform community leaders and members of the project objectives, activities, and preliminary results. Engagement was done in several study phases. In the first year of fieldwork, project researchers met with district officials to introduce the project objectives and planned activities. This engagement yielded valuable collaboration, particularly with regards to the district vector control focal person who engaged with project researchers in the field throughout the study. Community leaders from the 24 study villages were then introduced to the project and its potential benefits, with an emphasis on the planned interventions. The research team asked for the community leaders’ collaboration, especially in encouraging community member participation in project surveying activities. In the second year of fieldwork, before inception of the interventions, project researchers met with community leaders and members to again describe and answer any questions about the planned activities. Posters describing the project and interventions were provided to village leaders to display in a central public space. Community leaders were engaged to help in recruiting candidates for the local intervention staff positions (CHWs and larvicide applicators). At the beginning of the final year of fieldwork, district officials and community leaders were separately briefed on preliminary surveying and intervention findings, and once again presented with an opportunity to raise questions and provide feedback. 

### 2.3. Establishing the Study Population

#### 2.3.1. Process for the Random Selection of Villages

The random selection of villages was performed using updated geospatial data from the National Bureau of Statistics of Tanzania. The initial pool of candidate villages included all villages within the boundaries of six adjacent wards that had been previously selected by project researchers as a logistically feasible study area within Mvomero district. Before randomization, the decision was made to exclude villages that local leaders, including those from the district health office, deemed difficult to reach in the rainy season. 

**Figure 3:**
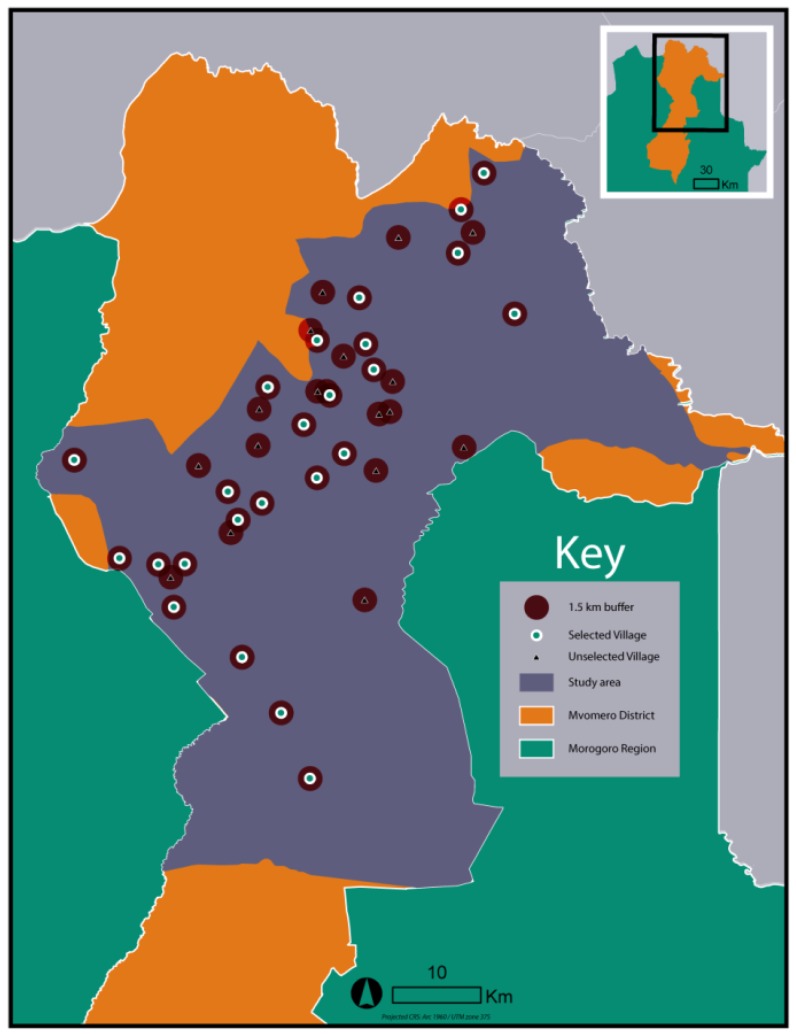
Randomly-selected run from the village selection procedure.

**Figure 4:**
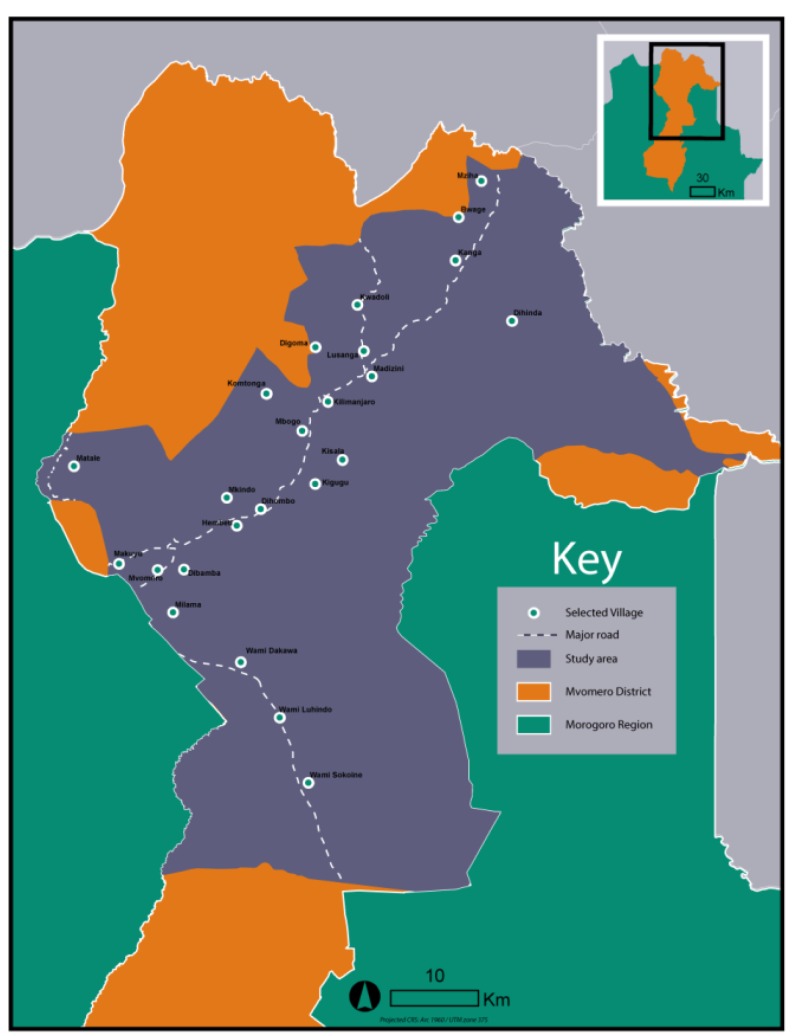
Map of selected villages.

The village randomization process was then performed using ArcGIS’ “Create Random Points” tool. The tool was constrained to generate points only from locations in the input shapefile, and to not generate two points within 3,000 meters of each other, in order to reduce the potential effects that an intervention in one village might have on a nearby village. The tool was run multiple times, generating multiple different random sets of villages. A representation of the randomly-selected run from among those generated during the procedure is provided in Figure 3. A map of the selected villages is shown in Figure 4. 

#### 2.3.2. Process for the Random Selection of Subvillages

Each village consists of a number of subvillages, which vary in the number of households contained therein. Subvillages were randomly selected for sampling within each village after excluding any subvillages that were considered by local leaders to be too difficult to reach in the rainy season. Sampling generally occurred in four subvillages per village, and occasionally in only three subvillages when the population among the subvillages in a particular village was deemed of a sufficient size for satisfying the household sampling procedure detailed below. No randomization process was done in villages containing only the required number of subvillages after excluding any unreachable subvillages. The number of households selected from each subvillage for surveying was determined by the proportion of eligible households relative to the total number of eligible households in the selected subvillages of a given village. 

#### 2.3.3. Process for the Random Selection of Households

The roster of eligible households among the selected subvillages of a village was generated from two hard-copy registries. Registries for a recent government distribution program of mosquito nets to households with children under five were used to capture households with children aged approximately one to five years. Because the net distribution program had occurred about one year before the date of randomization, households with children under one year of age at the time of randomization were not captured in the net distribution program registries. Rather, current vaccination registries obtained from local health facilities were used to capture households with infants below one year of age in the selected villages. The household information from the net distribution and vaccination registries were entered into an Excel file, merged, and randomized into an ordered list for each subvillage. 

For household surveying on knowledge, attitudes, and practices (KAP) related to malaria and other health issues, interviewers attempted to sample households from the randomized list in roughly sequential order, insofar as logistics permitted. As noted, the eligible study participants for the subsequent malariometric data collection were made up of the same households sampled in baseline KAP household surveying.

Households participating in the KAP survey and malariometric sampling were excluded from selection as a sentinel household for entomological evaluation. The three households in each village agreeing to participate in entomological surveying were not selected randomly, but rather were purposefully selected to capture a range of conditions including house construction type and features, proximity to the road, proximity to breeding sites, *etc.*

### 2.4. Description of Health Delivery Experiments Design and Implementation

The early detection and treatment experiment and the vector control health delivery experiment began and ran simultaneously from March through May in both 2012 and 2013. 

#### 2.4.1. Disease Management: Early Detection and Treatment Experiment

The disease management intervention was home-based, employing rapid diagnostic testing (RDT) and appropriate treatment by trained CHWs. Although use of RDTs is called for by the National Malaria Control Programme in Tanzania, at the time of the study RDTs were not widely available in the health facilities in the study area. Each village receiving this intervention was assigned one CHW, who was required to be a resident of the village and was recruited through a participatory process including the involvement of local village leaders and project staff. Selected CHWs received training chiefly led by two doctors closely involved in and knowledgeable about the project and its protocol. A project-appointed doctor supervised the appointed CHWs throughout the intervention period and liaised with local leadership and health facility staff to coordinate sensitization and referral processes. From March through May of each intervention year, CHWs made scheduled visits to each participating household approximately every two weeks. CHWs inquired about malaria treatment history in the past two weeks for each individual in the household. Axillary temperature was taken for all present participating household members regardless of reported illness. CHWs administered RDT to any participants with confirmed fever (*i.e.*, a temperature at or above 37.5 °C), as well as to any participants reporting fever in the past 2 days. All individuals with confirmed or reported fever in the course of the regular home visits received a referral to the nearest health facility, regardless of RDT result. Individuals with a positive RDT result were administered the proper ACT treatment by the CHW (ACTs were not dispensed to children under 5 kg or to pregnant woman according to standard treatment guidelines). Participating households were also encouraged to contact their CHW for an unscheduled visit should there be a case of reported fever in the household between scheduled visits. During any visit, if a participant received a positive RDT in two subsequent visits despite the administration of ACTs, CHWs were directed to contact the supervisor and refer the participant to a health facility for microscopic confirmation to rule out a false positive resulting from a previous recent infection. 

#### 2.4.2. Vector Control: Larviciding Experiment

Larviciding was done with the microbial agent of bacterial pathogens *Bacillus thuringiensis* var. israelensis (Bti) (Bactivec^®^, ValentBioSciences (VBS, Libertyville, IL, USA), CG formulation). The microbial larvicide *Bti* is effective against African malaria vectors, selective in targeted species, environmentally safe to non-target organisms, unlikely to result in resistance, and safe and simple for human handling. In large, open mosquito breeding habitats, the application of larvicide was done using Cifarelli sprayers. In smaller habitats, application was done by hand. The application dosage followed manufacturer instructions. 

Two local staff members were hired in each village selected for larviciding treatment. These local staff received extensive training and field observation from team entomologists as well as from a representative from VBS with experience deploying larvicide interventions in similar settings. A trained entomology supervisor was on-site throughout the three-month intervention period in each year to supervise, restock supplies as necessary, and conduct quality control spot checks. 

Local staff and other project team members collaborated with local leaders and consulted with community members to identify mosquito larvae habitats. Water body breeding habitats treated included rice paddies, ponds, shallow wells, streams, swamp areas, road-side canals, puddles, cement-lined pits, and temporary wetlands. Water bodies were classified by type according to established criteria. Local staff recorded larvae and pupae counts obtained through dipping at up to five capture stations using a 350 mL mosquito dipper per WHO standards for each water body both before initial larviciding and then again at regular intervals to measure the presence or absence of larvae before treatment and the change in larvae density over time after treatment. The number of capture stations per site was proportional to the breeding habitat. Local staff members were trained to categorize and count immature mosquitoes by stage of development (*i.e.*, early instars, late instars, and pupae). After being counted, the catch was returned to the site of capture. Larvicide was applied whenever vector larvae and/or pupae were found. Breeding habitats were evaluated at regular intervals to determine whether reapplication was necessary based on the above criteria. 

### 2.5. Statistical Methods and Power Calculations

Because young children are the most vulnerable to malaria, the primary endpoint of interest was parasitemia measured in children under the age of five years old in households within the villages. This section describes power calculations for this variable. Data were also collected for other age groups and endpoints—for example, anemia among pregnant women—but the study was specifically powered to test for changes in the proportion of young children with parasitemia. It was assumed that each eligible household contains on average 1.24 children under the age of five years. Parasitemia prevalence proportions were recorded in each village at three times during the study: (1) at baseline before the treatment assignment was introduced, (2) approximately 12 months after baseline, towards the end of the first round of interventions, and (3) approximately 24 months after baseline, towards the end of the second round of interventions. The data were collected at the same time each year during the height of the malaria season. 

The primary statistical objective was to estimate the magnitude of the effects over time for the four intervention groups. Baseline parasitemia prevalence proportions were established from measurements collected in the study area using methods outlined in Mboera *et al.* [24]. A sample of children from 24 villages (six villages from each treatment condition), 40 households per village, and an average of 1.24 children under the age of five years per household ensured that estimates of the non-zero changes in parasitemia (*i.e.*, from years 1 to 2 for Groups 2, 3 and 4 where the smallest assumed change from year 1 to 2 was 0.071 for Group 3 with an assumed prevalence proportion for year 1 = 0.354 and for year 2 = 0.283) were precise enough to produce 95% confidence intervals around these non-zero changes that did not overlap zero. Furthermore, given these sample sizes, the widths of confidence intervals around the change in proportions with parasitemia for all groups from years 1 to 2 and from years 2 to 3 were at most 0.10 where the largest assumed change from year 1 to 2 was 0.167 for Group 4 with an assumed prevalence proportion for year 1 = 0.354 and for year 2 = 0.187.

### 2.6. Safety and Ethical Considerations

All human subjects research components were documented in a human subjects research protocol approved by both the Duke University Institutional Review Board and the National Institute for Medical Research Ethical Review Committee before undertaking any fieldwork. The protocol also set forth strict requirements for maintaining participant confidentiality, and for the secure storage and transfer of study data. Permission to conduct the study was also granted by the Mvomero district authority and the community leaders in their respective study villages.

Participants signed consent forms for each research activity in which they were involved (*i.e.*, household surveying, malariometric and entomological sampling). For households participating in the disease management intervention, consent was also obtained from the household during the first CHW visit and subsequently from any participants on which an RDT was performed. Assent for minors was obtained from the parent or legal guardian. Larvicide staff complied with any directions from private land-holders who did not want larvicide applied on their land. 

The risks of the study were minimal in nature. Risks associated with finger pricks included momentary discomfort, bruising, infection, excess bleeding, clotting, and fainting. Mosquito light traps placed in homes participating in the entomological surveying also presented minimal risk. The risks to participants associated with the pyrethrum spray catch technique were minimal; pyrethrum is an insecticide which has been recognized for its low toxicity in humans and animals and limited environmental persistence for many years [26]. Larviciding with Bti presented minimal risk [27], and no adverse health effects have been found from human exposure to Bti [28]. 

The study stands to provide significant potential benefits to the study population. Potential benefits and preliminary findings were shared during community sensitization meetings moderated by research staff in each of the study villages. The early detection and treatment intervention also provided early detection and treatment of malaria cases, access to which is otherwise often limited by the money and time required for transportation, diagnosis, and treatment. More broadly, the findings from this project will inform policy and decision makers and other important stakeholders in malaria control.

### 2.7. Baseline Implementation

The baseline household study (Phase I) was completed over the course of approximately three weeks from mid-March through early April. The survey team consisted of the same NIMR researchers involved in the piloting and final review of the survey instrument. The first rounds of malariometric and entomological data collection took place roughly simultaneously from late April through mid-May 2011. Household KAP surveys were completed for a total of 962 randomly selected households (approximately 40 households per village) across all 24 villages. The total number of household members reported in the KAP across all of these households was 5,385. From among the households selected during the KAP surveying, 2,884 individuals from 720 of the selected households (75% of study households) participated in subsequent malariometric baseline surveying. Of these, 838 participants were children under-five years of age. FGDs were conducted in 12 of the 24 study villages, with half of the FGDs consisting of female participants only and half consisting of male participants only. Each FGD had from ten to twelve participants and lasted from one and a half to two h. An IDI was also conducted in each of the 24 villages. Researchers also collected baseline entomological data in April/May, June/July, July/August, and October 2011.

## 3. Results and Discussion

There is a striking gap between health innovations and their delivery to resource-poor settings. Most health improving interventions in low-income countries are not fully tested because they have not been evaluated under real world conditions. There is a growing recognition that increased research attention to implementation of health delivery is needed to improve health outcomes. This protocol presents the design of our study, which employed experiments and program evaluation methods to assess the effects and effectiveness of disease management and vector control strategies alone and in combination, implemented under realistic field conditions. The study encountered and addressed a number of challenges during implementation in the baseline year. The rosters used to determine household eligibility (*i.e.*, households with children under five) were often incomplete (and at times inaccurate) approximations of all eligible households, but were the most un-biased and complete records available to the researchers. Many of the households on the randomly-generated sampling list could not be included, mainly because either they could not be located, had moved away, or were not at home at the time of the surveyors’ visit. Despite eliminating from consideration villages which were hard to reach during the rainy season, heavy rains and distance (*i.e.*, distance of households from the main road and/or health facility) posed challenges to transport for both researchers and participants, which may have affected the ability of participating household members to present for the follow-up malariometric surveying at the identified central point. Another factor likely resulting in loss to follow-up in the baseline malariometric surveying is that it took place about a month after administering the household KAP survey due to logistical constraints. Finally, some household heads and/or members were wary of participating, particularly regarding a fear that the malariometric sampling contained a secret agenda to test participants for HIV/AIDS without the participants’ knowledge. Despite consenting during the first contact, a number of households withdrew from the study in subsequent visits. Community sensitization and greater engagement of local leaders were key to dispelling such misconceptions. 

## 4. Conclusions

This paper has described the protocol of our study which was designed to assess the effects and effectiveness of relevant, locally-feasible health delivery interventions for malaria control implemented under realistic field conditions. The design of the Mvomero project provides for two key contributions. First, it allows for the comparison of combinations of vector control and disease management interventions, allowing us to estimate the effect sizes of different intervention packages. Second, our implementation science approach contributes to one of the most neglected areas of research translation: how can new research results be effectively deployed in the field? Analysis of data from this study is currently ongoing. The results will contribute to an improved understanding of how to jointly optimize vector control and disease management strategies, building the knowledge base for evidence-based malaria control decision making by policy makers and public health practitioners in malaria endemic areas.
