# Correction: Kramer, R.A., *et al.* A Randomized Longitudinal Factorial Design to Assess Malaria Vector Control and Disease Management Interventions in Rural Tanzania. *Int. J. Environ. Res. Public Health* 2014*,* *11,* 5317–5332

**DOI:** 10.3390/ijerph110908622

**Published:** 2014-08-25

**Authors:** Randall A. Kramer, Leonard E. G. Mboera, Kesheni Senkoro, Adriane Lesser, Elizabeth H. Shayo, Christopher J. Paul, Marie Lynn Miranda

**Affiliations:** 1 Duke Global Health Institute, Duke University, 310 Trent Drive, Durham, NC 27710, USA; E-Mails: kramer@duke.edu (R.A.K.); adriane.lesser@duke.edu (A.L.); christopher.paul@duke.edu (C.J.P.); 2 Nicholas School of the Environment, Duke University, 9 Circuit Drive, Durham, NC 27708, USA; 3 National Institute for Medical Research, 2448 Barack Obama Drive, P.O. Box 9653, Dar es Salaam, Tanzania; E-Mails: lmboera@nimr.or.tz (L.E.G.M.); ksenkoro@nimr.or.tz (K.S.); eshayo@nimr.or.tz (E.H.S.); 4 School of Natural Resources and Environment, University of Michigan, 440 Church Street, Ann Arbor, MI 48109, USA; 5 Departments of Pediatrics and Obstetrics & Gynecology, School of Medicine, University of Michigan, Ann Arbor, MI 48109, USA

The authors wish to make the following corrections to their paper published in the *International Journal of Environmental Research and Public Health* [1]: 

Page 5326, line 24, the sentence “Larviciding was done with the microbial agent of bacterial pathogens *Bacillus thuringiensis* var. israelensis (Bti) (Bactivec^®^, ValentBioSciences (VBS, Libertyville, IL, USA), CG formulation).” should read “Larviciding was done with a granular formulation (VectoBac® CG) of the bacterial larvicide (BL) *Bacillus thuringiensis* var. *israelensis* (*Bti*) strain AM65-52, Valent BioSciences Corporation (VBC, Libertyville, IL, USA)”.

The authors would like to apologize for any inconvenience caused to readers by these changes.
